# Supplementary material for: Global change in brain state during spontaneous and forced walk in Drosophila is composed of combined activity patterns of different neuron classes
Source: eLife. 2023 Apr 17;12:e85202. doi: 10.7554/eLife.85202 (PMC10168698; doi:10.7554/eLife.85202)
Supplement: Supplementary file 1. [file elife-85202-supp1.docx]

| **Short name** | **Description** | **Examples of matching (Flycircuit) neurons** | **present in Gal4 line** (detected in ≥ 5 flies) |
| --- | --- | --- | --- |
|  |  |  |  |
| **Antennal lobe and Mushroom body** | | | |
| AL | Whole antennal lobe | Gad1-F-000394; Gad1-F-100601; Gad1-F-800129; VGlut-F-900126 | Nsyb , GMR57C10 , Vglut , Gad |
| PN | antennal lobe projection neuron | VGlut-F-500486 | Nsyb , GMR57C10 , Cha , Vglut , Gad |
| MultiGl | Whole antennal lobe and PN-like projections | Trh-F-600017 | TH , Trh , TDC |
| PNv | antennal lobe projection neuron, ventral tract of the lateral horn | VFB_00101133 | Nsyb , GMR57C10 , Cha , Vglut , Gad |
| PN-KC | Antennal lobe projection neuron and kenyon cell in the same component |  | Nsyb , GMR57C10 , Cha, Vglut |
| KCab | Alpha-Beta Kenyon cell | Cha-F-300226; Cha-F-100049; fru-F-000026; Vglut-F-100284; Gad1-F-100014; | Nsyb , GMR57C10 |
| KCapbp | Alpha’-Beta’; Kenyon cell | Gad1-F-100024; Trh-F-200069; | Nsyb , GMR57C10 |
| KCg | Gamma kenyon cells | fru-F-000006; Vglut-F-100359; Gad1-F-100021; | Nsyb , GMR57C10 |
| Beta1Betap1 | Beta1 and/or Beta’1 mushroom body compartment | PAM10(B1)_L (FlyEM-HB:1328522741) [VFB_jrchk385] | TH |
| Beta2Betap2 | Beta2 and/or Beta’2 mushroom body compartment | PAM02(B’2a)_L (FlyEM-HB:1295566429) | Nsyb, Gad , TH |
| **Gamma1** | **Gamma1 mushroom body compartment** | **PPL1-gamma1-pedc** | **TH** |
| **Gamma2** | **Gamma2 mushroom body compartment** | **PPL1-gamma2-alpha’1** | **TH** |
| **Gamma3** | **Gamma3 mushroom body compartment** | **MBON-γ3, PAM-γ3** | **Gad, TH** |
| **Gamma4** | **Gamma4 mushroom body compartment** | **PAM-γ4** | **TH** |
| **Gamma5** | **Gamma5 mushroom body compartment** | **PAM-γ5** | **TH** |
| SLP-Alpha | Alpha or Alpha&apos; lobe with projection through the superior lateral neuropil | VGlut-F-500002 | Vglut |
| **Alpha1** | **Alpha1 mushroom body compartment** | **PAM-alpha1** | **TH** |
| **Alpha2** | **Alpha2 mushroom body compartment** | **PPL1-alpha’2alpha2** | **TH** |
| **Alpha3** | **Alpha3 mushroom body compartment** | **PPL1-alpha3** | **TH** |
| **Alphap3** | **Alpha’3 mushroom body compartment** | **PPL1-alpha’3** | **TH** |
| **Superior neuropil** | | | |
| SCLtract | Tract linking both superior clamp | Trh-F-200082,Trh-F-100051 | Trh |
| CLvert | Lateral part of the superior clamp | TH-F-000023 | Nsyb, TH |
| CL? | Interrogation point surrounding the pedonculus, | Trh-F-300074,Trh-M-700081, DNp32 | TH , Trh |
| CL-LH | Surrounds the lateral horn from the medial and ventral directions | Trh-F-200047 | Trh , TDC |
| CL | Other shapes at the level of the clamp | Gad1-F-700550,Gad1-F-800055 | Nsyb, GMR57C10 , Gad, TH, TDC |
|  |  |  |  |
| SMPm | Medial part of the superior medial protocerebrum | TH-F-000021; VGlut-F-700286, Cha-F-300251 | Nsyb, GMR57C10, Cha, Vglut, Gad , TH, TDC |
| PPL-SMP | ventral lateral part of the superior medial protocerebrum, with tracts coming from a posterior lateral cell cluster | TH-F-000019,TH-F-000018,TH-F-000046 | TH |
| SMPl-SIP | Superior intermediate protocerebrum and lateral part of the superior medial protocerebrum | Cha-F-000221,Cha-F-300154, TH-F-300056 | Nsyb , GMR57C10 , Cha , Gad, TH , TDC |
| SIP-SMPd | Superior intermediate protocerebrum and dorsal part of the superior medial protocerebrum | fru-F-800063 | Nsyb |
| SIP-FB | Superior intermediate protocerebrum and dorsal layer of the fan-shaped body | Trh-F-100015; | Nsyb, GMR57C10 , TH, Trh |
| FB-SN | Broad innervaion of the superior neuropil, and fan-shaped body | OA-VMP3, OA-VPM4 | TDC |
| SLP-SMPproj | Large SMP neuron projecting to ventral regions | Trh-F-700011,Trh-F-000083 | Nsyb , GMR57C10, TH , Trh |
| SLP-SMP | Superior lateral protocerebrum and superior medial protocerebrum | Trh-F-500176, DNp25 | Trh |
| SLP | Superior lateral protocerebrum only | TH-F-100046 | Nsyb, GMR57C10, TH , TDC |
| LH-SLP | Lateral horn and superior lateral protocerebrum | Gad1-F-900346,Gad1-F-600340 | Nsyb , Gad |
| **Central complex** | | | |
| FBcol | Fan shaped body columns | Tdc2-F-100009; Tdc2-F-300026; Tdc2-F-300001; Tdc2-F-200011; Tdc2-F-100062; Tdc2-F-100016; Gad1-F-900245; Gad1-F-800329; Gad1-F-500513; Gad1-100157 | Nsyb , GMR57C10, Vglut , Gad, TDC |
| FBlayv | Ventral layer of the fan-shaped body | TH-M-300065 | Nsyb, TH |
| FBlaym | Medial layer of the fan-shaped body | TH-F-200055 | Nsyb , TH |
| FBlayd | Dorsal layer of the fan-shaped body | TH-F-200054; Trh-F-300036; Trh-F-400062 | Nsyb, TH , Trh |
| NO | Nodulus or noduli | Cha-F-100429 | Nsyb |
| PB | Protocerebral bridge only | Vglut-F-800282; Vglut-F-600784; Vglut-F-600229; Gad1-F-600267; Gad1-F-100361; Gad1-F-100593; Vglut-F-000156; Vglut-F-100064 | Nsyb , Vglut , Gad |
| PBfull | Components with full protocerebral bridge | Cha-F-900016, Cha-F-200148 | Nsyb , GMR57C10 , Cha |
| **PB-DA** | **Protocerebral bridge and two dots (maybe cell bodies) at the top of the trachea** | **TH-F-000048** | **TH** |
| BU-PBl-EB | Bulb, ellipsoid body and lateral part of the protocerebral bridge | Gad1-F-900445 | Nsyb , Gad |
| PB-EB | EB-radial and PB glomeruli | Cha-F-500009 | Nsyb , GMR57C10 , Cha, Gad |
| EB | Ellipsoid body rings | Trh-F-300095; Cha-F-800146 | Nsyb, GMR57C10, Cha, Vglut , Trh |
| **EB-DA** | **Ellipsoid body and lateral accessory lobe** | **TH-F-100001** | **TH** |
| AOTU-BU | Anterior optic tubercule and bulb | Gad1-F-200712, VGlut-F-400630 | Nsyb , GMR57C10 , Cha, Vglut, Gad |
| **Posterior neuropil** | | | |
| IB | Inferior bridge |  | Nsyb, Gad |
| ATL | Antler | adult antler neuron 031 | TH, Trh |
| M-Omega | Posterior ensemble forming an M dorsally and an omega ventrally | TH-F-300078 | TH |
| SPS | Superior posterior slope | VGlut-F-900089, VGlut-F-800136,Cha-F-800003,Gad1-F-900039 | Nsyb , GMR57C10, Cha , Vglut , Gad |
| IPS-Y | inverse Y shape in the posterior slope | DNb02? | Nsyb , GMR57C10 , Cha, Vglut, Gad |
| LAL-PS | lateral accessory lobe and posterior slope | DNb01? | Nsyb , GMR57C10 , Cha, Vglut , Gad |
| **PPM2-LW** | **PPM2-LAL-We L,R** | **TH-F-000000,TH-F-000015,TH-F-000016** | **TH** |
| **PPM2-VI** | **PPM2-VMNP-INP L,R** | **TH-F-000007,TH-F-300058** | **TH** |
| **Lateral neuropil** | | | |
| WPENb | antennal mechanosensory and motor center and/or Wedge, in the posterior lateral protocerebrum and posterior connection to opposite side | VGlut-F-200005,WPNb, WPNB3#5 (FAFB:4271367) [VFB_001011lp] | Vglut , Gad |
| AMMC-PLP | antennal mechanosensory and motor center and/or Wedge and branch in the posterior lateral protocerebrum | VGlut-F-400269 | Nsyb , Cha , Vglut, Gad |
| AMMC-WE | antennal mechanosensory and motor center and/or Wedge | VGlut-F-000138, VGlut-F-400586 | Nsyb , GMR57C10 , Vglut , Gad |
| **WE-DA** | **Wedge with two branches forming a large V** | **TH-F-200127,TH-F-000024** | **TH** |
| AVLPonlyproj | Lowest medial part of the anterior ventral lateral protocerebrum projecting ventrally | Cha-F-700097 |  |
| AVLPprojm | Lowest medial part of the anterior ventral lateral protocerebrum projecting ventrally | Gad1-F-500762, Cha-F-400059; Gad1-F-000013 | Nsyb , GMR57C10, Gad |
| AVLPprojl | Lowest lateral part of the anterior ventral lateral protocerebrum projecting ventrally | Cha-F-800125 | Cha |
| AVLPm | anterior ventral lateral protocerebrum medial part | Gad1-F-900529, Cha-F-800062,Trh-F-400039,Trh-F-400070; Vglut-F-200405*; Vglut-F-900122; Cha-F-400237; Cha-F-200299; Gad1-F-500279 | Nsyb , GMR57C10 , Vglut, Gad , TH, Trh, TDC |
| AVLPd | anterior ventral lateral protocerebrum dorsal part | Cha-F-000424 | Nsyb |
| AVLPshell | anterior ventral lateral protocerebrum surface | Trh-F-100082 | TH, Trh |
| AVLPsmear | anterior ventral lateral protocerebrum anterior part |  | Nsyb |
| VLPl | Ventro-lateral protocerebrum most lateral part | Gad1-F-900096, Vglut-F-500616,Cha-F-800087 | Nsyb , GMR57C10 , Cha , Vglut, Gad |
| PLP-LH | Posterior lateral protocerebrum to the basis of the lateral horn | Gad1-F-500325 | Nsyb |
| PLP | Posterior lateral protocerebrum | Gad1-F-800092 | Nsyb, Gad |
| **Ventral neuropil** | | | |
| PI | Pars intercerebralis | Trh-F-100040, Trh-M-000056 | Nsyb , GMR57C10 , TH , Trh |
| PI-PRW | Pars intercerebralis connected to Prow | VGlut-F-600158 | Nsyb , GMR57C10 , Gad |
| PRW | Prow | TH-M-000037 | Nsyb , GMR57C10 , Vglut, Gad , TH |
| PRW-SLP | Prow and superior lateral protocerebrum | Gad1-F-600213, Cha-F-200258; fru-F-000133; Gad1-F-600213; Trh-F-100091 | Nsyb , GMR57C10 |
| PENP-CL | periesophageal neuropils and clamp | mALD3_L (FlyEM-HB:822708945) | Nsyb , GMR57C10, Cha , Gad |
| GNGvw | gnathal ganglia medial and lateral | Cha-F-400186 | Cha |
| GNGm | gnathal ganglia medial | Cha-F-300235 | Nsyb , GMR57C10 |
| GNGml | gnathal ganglia medial-lateral | Cha-F-400159 | Nsyb , GMR57C10, Cha, Gad |
| GNGl | gnathal ganglia lateral | Cha-F-400146 | Nsyb, Cha |
| GNG-AMMC | gnathal ganglia and on the opposite side antennal mechanosensory and motor center and posterior lateral protocebrum | VGlut-F-600685 | Nsyb, Vglut |
| GNGva | ventral anterior part of the gnathal ganglia |  | Nsyb |
| vaCells | ventral anterior cells | TH-F-100049 | TH , Trh |
| **Optic lobe** | | | |
| OL | Optic lobe; mostly medulla and lobulla |  | Nsyb, GMR57C10 , Cha , Vglut , Gad |
| LOP | Lobulla plate | Cha-F-600161 | Cha, TDC |
| OL-FB | Optic lobe to central regions including the fan-shaped body |  | TH |
| OL-PENP | Optic lobe and periesophageal neuropils | Tdc2-F-200056 | Nsyb , Gad , Trh , TDC |
| OL-PLP | Optic lobe and posterior lateral protocerebrum | Cha-F-000316 | Nsyb , Cha, Gad |
| OL-WE | Optic lobe and wedge | TH-F-300030 | TH |
|  |  |  |  |
